# Effect of locomotor preference on the evolution of mitochondrial genes in Bovidae

**DOI:** 10.1038/s41598-024-63937-5

**Published:** 2024-06-05

**Authors:** Lupeng Shi, Xibao Wang, Xiufeng Yang, Tianshu Lyu, Lidong Wang, Shengyang Zhou, Yuehuan Dong, Xiaoyang Wu, Yongquan Shang, Honghai Zhang

**Affiliations:** College of Life Sciences, Qufu Normal University, Qufu, Shandong China

**Keywords:** Bovidae, Locomotor preference, Habitat types, Evolution, mtDNA, Evolution, Zoology

## Abstract

Locomotor preferences and habitat types may drive animal evolution. In this study, we speculated that locomotor preference and habitat type may have diverse influences on Bovidae mitochondrial genes. We used selection pressure and statistical analysis to explore the evolution of mitochondrial DNA (mtDNA) protein-coding genes (PCGs) from diverse locomotor preferences and habitat types. Our study demonstrates that locomotor preference (energy demand) drives the evolution of Bovidae in mtDNA PCGs. The habitat types had no significant effect on the rate of evolution in Bovidae mitochondrial genes. Our study provides deep insight into the adaptation of Bovidae.

## Introduction

The mitochondrial genome is a circular DNA molecule that plays a key role in aerobic respiration. Therefore, ensuring mitochondrial function is essential for maintaining the metabolic activity of the animals. Bovidae mtDNA contains 13 PCGs. These 13 mtDNA PCGs play important roles in oxidative phosphorylation (OXPHOS)^1^. The mitochondrial genome also includes 22 transfer RNA genes, 2 ribosomal RNA genes, and 1 control region. These genes form two strands (heavy strand and light strand) that make up the mitochondrial genome. Mitochondria provide approximately 95% of the total energy to eukaryotic cells and serve as the energy factories of eukaryotic cells^2^. The energy produced by mitochondria can maintain body temperature and activity^3^. In addition, mitochondrial genes are characterized by a simple structure and maternal heredity^4–7^. Mitochondrial genes are diffusely used for adaptive evolution and phylogenetic relationships among organisms^8^. In summary, a calculation of the selection pressures on mtDNA can provide novel insight into the evolution of mtDNA.

Bovidae is an ecologically important taxonomic family that plays a vital role in nature. The habitats of the members of Bovidae (known as bovids) are varied, including forests, grasslands, and deserts^9,10^. The morphological structure and physiological characteristics of bovids are different in different habitats. The evolution of Bovidae is modeled by several mechanisms, including temperature, vegetation physiognomy, climate, and radiation^11^. For example, Kappelman found that bovids living in plains have laterally expanded femoral heads, whereas bovids living in forests have spherically shaped femoral heads^12^. To survive well, bovids require diverse amounts of energy in diverse habitats to meet their needs. In addition, according to the International Union for the Conservation of Nature (IUCN), some bovids have developed physiological and morphological adaptations for climbing in steep rocky areas. For example, the Pleistocene mountain goat (*Oreamnos harringtoni*) has evolved slightly more robust hind limbs to adapt to steep slopes^13^. Studies of ruminants by Alexandre Hassanin et al. found that ruminants living in mountainous areas have a unique molecular adaptation of ATPase, which optimizes OXPHOS efficiency^14^. Therefore, bovids require tremendous energy to adapt to climbing. Some studies also found that bovids in open, flat areas have developed rapid locomotion, whereas ruminants living in forests can jump frequently^12,15^. Therefore, different animals require different amounts of energy to adapt to different locomotor preferences. Research has demonstrated that habitat type affects mitochondrial gene evolution^16,17^. Therefore, we supposed that the habitat type and locomotor preferences of bovids might have influenced the evolution of their mitochondrial genome.

The non-synonymous/synonymous substitution rate (dN/dS) is often used to calculate the evolution of 13 mtDNA PCGs^18,19^. Thus, we calculated the rate of evolution of Bovidae mitochondrial genes using dN/dS. We aimed to determine whether Bovidae mtDNA evolutionary rate is related to habitat type and locomotor preference. Our research can provide deep insight into the adaptation and evolution of bovids.

## Materials and methods

### Species sample and mtDNA sequence data

According to the criteria of IUCN, we selected 48 bovids from different habitats: 22 forest (FG), 21 grassland (GG), and 5 desert (DG) (Table S1). Mitochondrial genomes were downloaded from NCBI (https://www.ncbi.nlm.nih.gov/). According to the IUCN description of species' habitats, Some Bovidae species forage for or avoid predators in steep rocky areas. Therefore, we classified the 48 bovid into climbing (CG) and non-climbing (NCG) groups (Table S1). In the IUCN, the habitats of climbing species include rocky areas, whereas the habitats of non-climbing species do not include rocky areas. All in all, the 48 bovids did not overlap with each other in terms of habitat types (forest, grassland, and desert) or locomotor preferences (climbing and non-climbing). In other words, a bovid can occupy only one of the three habitat types (forest, grassland, or desert) and one of the two locomotor preferences (climbing and non-climbing) as per the IUCN.

### Phylogenetic construction

The 13 mtDNA PCGs were extracted from Bovidae mitochondrial genome and aligned using MUSCLE software^20^. In this study, 14 sequence datasets, each containing PCG and 13 PCGs (13 PCGs concatenated into one sequence), was obtained (Table S1). *Equus asinus* (NC_001788.1) was used as the outgroup and the Bayesian inference (BI) was applied to construct phylogenetic trees. The PhyloSuite software was used to select the optimal model (GTR + F + I + G4)^21^.

The phylogenetic relationship was determined by comparing the 13 mtDNA PCGs based on the BI. Sequence files for constructing the phylogenetic tree are shown in Table S2. We performed the concatenated matrix with four simultaneous Markov Chain Monte Carlo (MCMC) chains, sampling one tree every 1000 generations and running it for 20 million cycles. The iTOL online software was used to visualize the phylogenetic trees.

### Selection pressure analyses

The CodeML program in PAML package was used to evaluate dN/dS (ω)^22^. We used MAFFT software to align the protein-coding genes^23^. We used Gblocks to remove the unaligned areas^24,25^. Finally, the results of sequence alignment are used for selection pressure analysis. If any ω were unexpectedly small or large (approaching zero indefinitely or tending toward infinity), we labeled them as “NA” in Table S3; “NA” was not included in our analyses. These extreme ω occur mainly owing to high saturation or low substitution values^26^. The specific reasons mainly include: (1) most of the genes are not prone to accumulate nucleotide substitutions; (2) PCGs of mitochondrial genes are highly conserved and not prone to frequent mutations^27,28^.

To identify the positive selection genes of bovids in different habitats and locomotor preferences, we compared the ω of each mtDNA PCG of different groups using the branch model (NSsites = 0, one-ratio model [M0], and two-ratio model [M2]. To identify whether positive selection appeared in diverse groups, FG, GG, DG, and CG were used as the foreground branches, and the branch-site model (NSsites = 2, one-ratio model [M0], and two-ratio model [M2]) was used for the analysis.

### Statistical analysis

Analysis of variance (ANOVA) was used to calculate the statistical significance of the ω among different habitat types (forest groups, FG; grassland groups, GG; and desert groups, DG). The Wilcoxon test was used to calculate the statistical significance of the ω among diverse locomotor preferences (climbing groups, CG; non-climbing groups, NCG). To determine the phylogenetic impact, we used phylogenetic ANOVA^29,30^. The code for statistical analysis is shown in Table S4.

### Institutional review board statement

Because non-invasive samples were collected, ethical review were waived.

## Results

### Phylogenetic analyses

**Figure 1:**
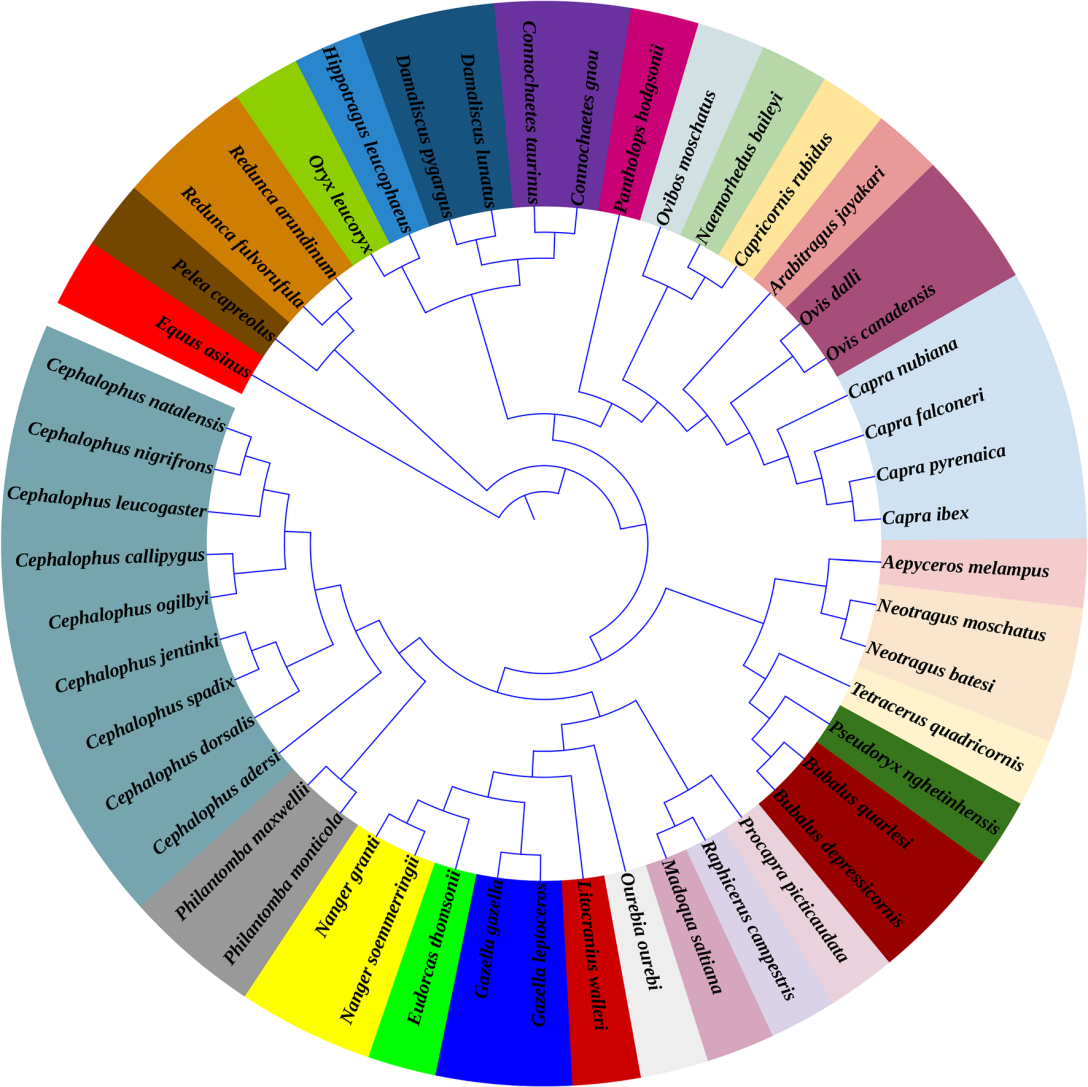
The BI phylogenetic tree of 48 Bovidae species based on 13 PCGs of mitochondrial genomes. Different colors represent different genera.

We used 13 PCGs of 48 bovids for the phylogenetic analysis (Fig. 1). The average potential scale reduction factor (PSRF) for parameter values is 1.005. According to previous research, when runs converge, the PSRF should approach 1^31,32^. Therefore, the quality of the phylogenetic tree that we built is good. Although the topological structure of this tree is different from that of other studies, it is generally consistent^33–35^. There are two main reasons for this difference: (1) the selected molecular markers are different. Zhao et al. used DNA control region, whereas we used the whole mitochondrial genome; (2) fossil calibration may result in subtle changes in the topology. Therefore, this phylogenetic topology was credible for subsequent analyses.

### Free ratio model analysis

CodeML was used to calculate the ω. ω > 1 represents positive selection, ω < 1 represents purifying selection, and ω = 1 represents neutral selection. All the ω were < 1 (provided in Table S3), explaining that 48 bovids were under purifying selection.

### Positive selection analysis

To evaluate ω of the Bovidae mitochondrial genomes under the three habitat types (forest, grassland, and desert), we used the branch and branch-site models. Four rapid evolutionary genes were discovered by branch model in the DG (p < 0.05), namely ND2 (FG: ω, 0.0361561; GG: ω, 0.0210023; DG: ω, 0.0379897), ND3 (FG: ω, 0.0191199; GG: ω, 0.0129913; DG: ω, 0.107196), ND4 (FG: ω, 0.0304507; GG: ω, 0.022008; DG: ω, 0.0443077), and ND5 (FG: ω, 0.0419007; GG: ω, 0.0427425; DG: ω, 0.0653745) (Table 1). The branch-site model revealed that five genes (ATP6, ND2, ND4, ND5, and COX3) had positively selected sites in the FG, five genes (ND1, ND2, ND4, ND5, and Cytb) had positively selected sites in the GG, and six genes (ATP6, ND2, ND4, ND5, COX1, and COX3) had positively selected sites in the DG (Tables 2, 3, and 4).

**Table 1:** The rapidly evolving genes of 48 Bovidae species from different habitat types based on the branch model.

Gene	2ΔlnL	p-value	The ω of FG	The ω of GG	The ω of DG
ATP6	5.259896	0.153725949	0.0413113	0.0279492	0.0485421
ATP8	5.530752	0.136810833	0.205611	0.222378	0.151424
ND1	2.09604	0.552714314	0.0138016	0.0107604	0.0144459
ND2	12.477764	0.005913519	0.0361561	0.0210023	0.0379897
ND3	12.164646	0.006839917	0.0191199	0.0129913	0.107196
ND4	11.291604	0.010249178	0.0304507	0.022008	0.0443077
ND4L	3.526226	0.317376592	0.0206484	0.0238195	0.0089278
ND5	12.788808	0.005116309	0.0419007	0.0427425	0.0653745
ND6	1.786086	0.617967449	0.0208479	0.0190476	0.026773
COX1	4.43313	0.218333058	0.00537933	0.00636838	0.00998793
COX2	2.8011	0.423318869	0.00500671	0.00772302	0.0133358
COX3	2.773548	0.427872919	0.0215251	0.016253	0.0141903
Cytb	5.984424	0.112370495	0.0175695	0.0236066	0.0304319

*ω* evolution rate, *FG* forest species, *GG* grassland species, *DG* desert species.

**Table 2:** Positively selected sites for 13 mtDNA PCGs in the FG (foreground branch) based on the branch-site model.

Gene	Model	2ΔlnL	p-value	Positively selected sites (BEB analysis)
ATP6	Model A null model	0	1	51 V 0.984*; 61 P 0.701; 183 L 0.883
ATP8		0.65157	0.419551922	
ND1		0	1	
ND2		0	1	246 S 0.904
ND3		0	1	
ND4		0	1	54 L 0.587; 81 H 0.778; 87 N 0.668; 113 F 0.600; 163 S 0.990**; 178 P 0.529
ND4L		0	1	
ND5		0.39682	0.528736128	500 V 0.944
ND6		0.23895	0.624965511	
COX1		2.448948	0.117603662	
COX2		0	1	
COX3		0	1	39 T 0.558
Cytb		0	1	

*BEB analysis* means Bayes empirical Bayes analysis. *FG* forest species. *means BEB > 0.95; **means BEB > 0.99.

**Table 3:** Positively selected sites for 13 mtDNA PCGs in the GG (foreground branch) based on the branch-site model.

Gene	Model	2ΔlnL	p-value	Positively selected sites (BEB analysis)
ATP6	Model A null model	2E–05	0.996431764	
ATP8		0	1	
ND1		0	1	33 L 0.978*; 309 S 0.808
ND2		0	1	214 I 0.585
ND3		0	1	
ND4		0	1	21 N 0.672; 360 V 0.611
ND4L		2.521884	0.112276372	
ND5		0	1	199 I 0.959*; 485 S 0.927; 533 T 0.702; 565 I 0.550; 596 I 0.948
ND6		0	1	
COX1		0	1	
COX2		0	1	
COX3		0	1	
Cytb		0	1	107 L 0.980*; 114 I 0.916; 210 S 0.973*

*BEB analysis*: Bayes empirical Bayes analysis, *GG* grassland species. *means BEB > 0.95.

**Table 4:** Positively selected sites for 13 mtDNA PCGs in the DG (foreground branch) based on the branch-site model.

Gene	Model	2ΔlnL	p-value	Positively selected sites (BEB analysis)
ATP6	Model A null model	0	1	7 A 0.732
ATP8		0	1	
ND1		0	1	
ND2		0	1	139 M 0.560; 217 T 0.968*
ND3		0	1	
ND4		0	1	93 L 0.834; 358 M 0.905
ND4L		0	1	
ND5		0	1	36 I 0.647; 434 P 0.640
ND6		0	1	
COX1		0	1	462 L 0.572
COX2		0	1	
COX3		0	1	116 H 0.784
Cytb		0	1	

*BEB analysis*: Bayes empirical Bayes analysis, *DG* desert species. *means BEB > 0.95.

Locomotor preference may impose diverse influences on the Bovidae mtDNA. To test this, we used branch and branch-site models to compare the ω of protein-coding genes in the CG and NCG. Ten rapid evolutionary genes were discovered by branch model in the CG (p < 0.05), namely ATP6 (CG: ω, 0.108253; NCG: ω, 0.028488), ND2 (CG: ω, 0.0515474; NCG: ω, 0.0267577), ND3 (CG: ω, 0.0430146; NCG: ω, 0.0170811), ND4 (CG: ω, 0.0387976; NCG: ω, 0.0257132), ND4L (CG: ω, 0.0502962; NCG: ω, 0.0216764), ND5 (CG: ω, 0.0677209; NCG: ω, 0.045852), COX1 (CG: ω, 0.0150486; NCG: ω, 0.00486651), COX2 (CG: ω, 0.0234107; NCG: ω, 0.00540546), COX3 (CG: ω, 0.0493184; NCG: ω, 0.0147064), and Cytb (CG: ω, 0.0404504; NCG: ω, 0.0205027) (Table 5). The branch-site model showed that ATP6, ATP8, ND1, ND2, ND4, ND5, COX3, and Cytb had positively selected sites in CG (Table 6).

**Table 5:** The rapidly evolving genes of 48 Bovidae species with different locomotor preference based on the branch model.

Gene	2ΔlnL	p-value	The ω of CG	The ω of NCG
ATP6	32.249668	1.3558E–08	0.108253	0.028488
ATP8	0.475004	0.490694057	0.234931	0.166745
ND1	3.597486	0.057867018	0.0206553	0.0121761
ND2	10.67065	0.001088489	0.0515474	0.0267577
ND3	5.830352	0.015751971	0.0430146	0.0170811
ND4	4.66782	0.030732914	0.0387976	0.0257132
ND4L	4.026272	0.044796827	0.0502962	0.0216764
ND5	8.044672	0.004563768	0.0677209	0.045852
ND6	0.976458	0.323074836	0.0295556	0.0213581
COX1	12.336058	0.000444292	0.0150486	0.00486651
COX2	12.977638	0.000315233	0.0234107	0.00540546
COX3	17.47922	2.90465E-05	0.0493184	0.0147064
Cytb	11.594512	0.000661467	0.0404504	0.0205027

*ω*: evolution rate. *CG* climbing group, *NCG* non-climbing group.

**Table 6:** Positively selected sites for 13 mtDNA PCGs in the CG (foreground branch) based on the branch-site model.

Gene	Model	2ΔlnL	p-value	Positively selected sites (BEB analysis)
ATP6	Model A null model	0	1	7 A 0.633; 20 V 0.696; 51 V 0.997**; 109 A 0.699; 120 M 0.966*; 125 L 0.769
ATP8		0.668176	0.41368826	10 L 0.957*; 23 I 0.953*; 41 K 0.536
ND1		0	1	249 M 0.727
ND2		0	1	5 I 0.991**; 37 N 0.844; 83 T 0.680; 265 T 0.978*; 311 E 0.986*
ND3		0	1	
ND4		0	1	87 N 0.859; 93 L 0.702; 163 S 0.882
ND4L		0	1	
ND5		0	1	18 M 0.644; 25 S 0.624; 36 I 0.564; 86 V 0.964*; 189 L 0.695; 491 I 0.742; 511 Y 0.734; 557 I 0.562
ND6		0.248106	0.61841187	
COX1		0	1	
COX2		0	1	
COX3		0	1	39 T 0.954*; 42 M 0.682; 152 A 0.989*
Cytb		0	1	120 F 0.511; 187 A 0.812; 225 I 0.837; 287 V 0.502

*BEB analysis* means Bayes empirical Bayes analysis, *CG* climbing group. *means BEB > 0.95; **means BEB > 0.99.

### Statistical analysis

**Figure 2:**
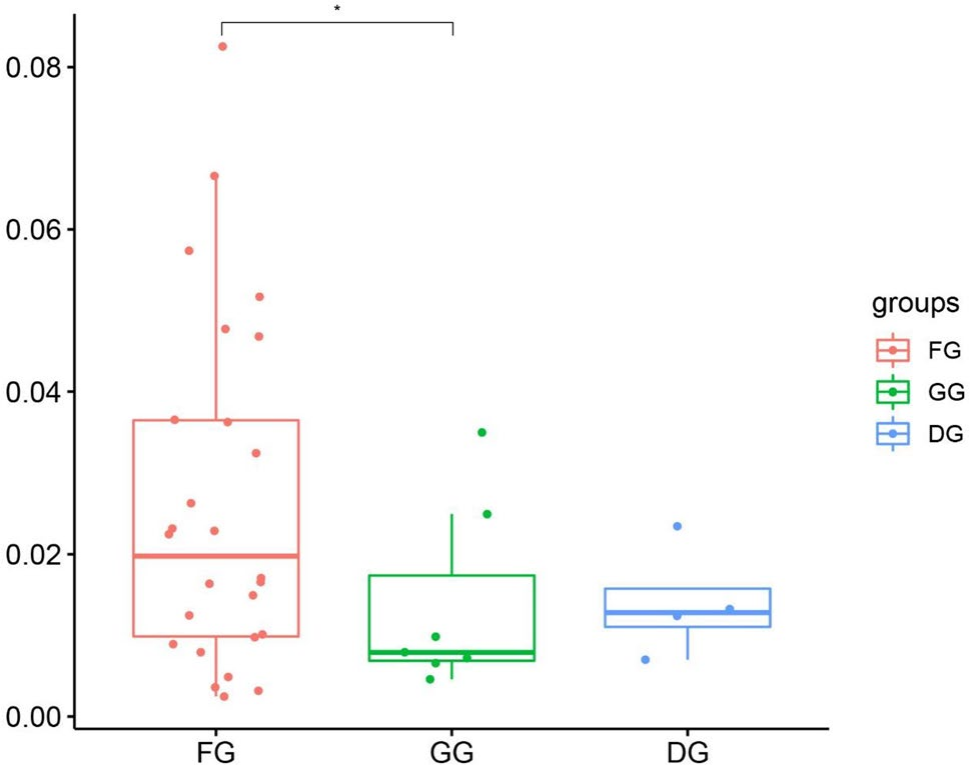
Comparisons of ω among 48 bovid species from different habitat types on ND1 (*p < 0.05).

**Figure 3:**
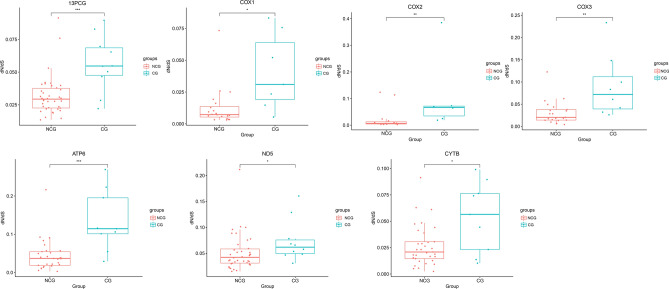
Comparisons of ω on 13 PCGs, COX1, COX2, COX3, ATP6, ND5, and Cytb (*p < 0.05; **p < 0.01; ***p < 0.001) among 48 bovid species with different locomotor preference.

ANOVA revealed that the ω of ND1 were significantly lower in the GG than in the FG (Fig. 2). The other mtDNA PCGs were not significantly different among the FG, GG, and DG. In addition, the Wilcoxon test revealed that the ω of the 13 PCGs, COX1, COX2, COX3, ATP6, ND5, and Cytb were significantly lower in the NCG than that in the CG (Fig. 3). The other mtDNA PCGs were not significantly different between the CG and NCG.

**Figure 4:**
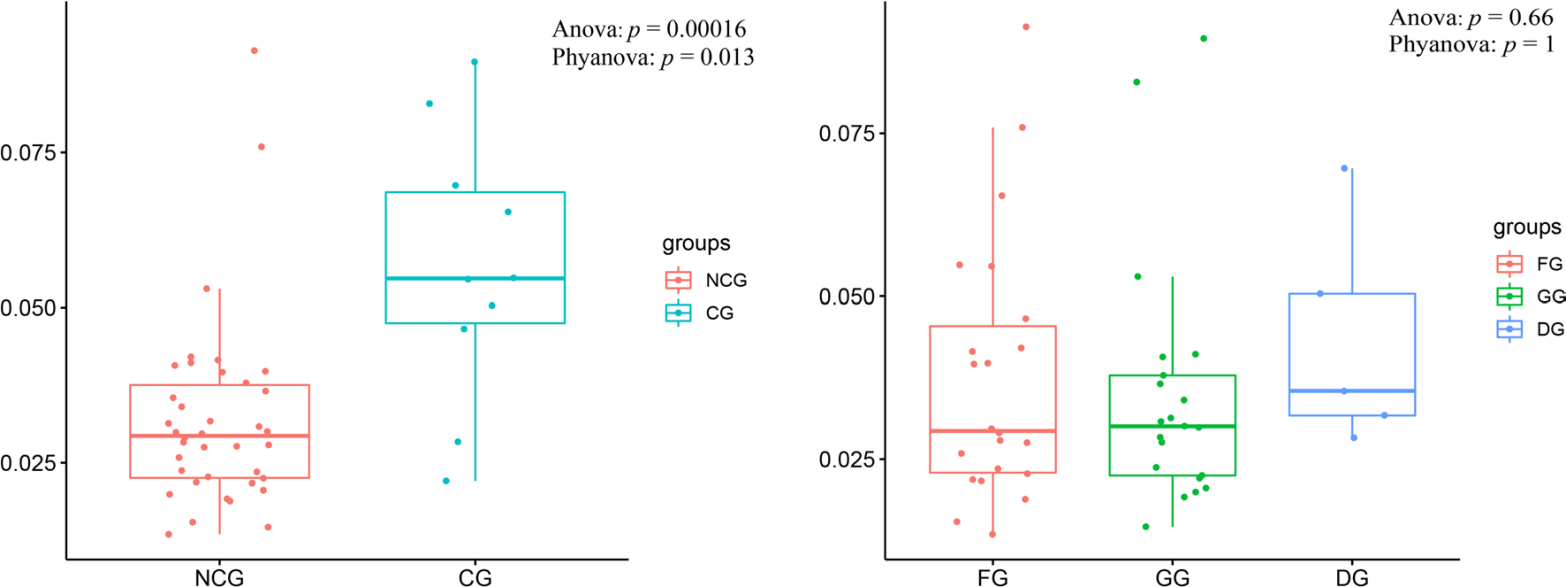
Phylogenetic ANOVA of ω among 48 bovid species of different groups.

Phylogenetic ANOVA revealed that the ω of 13 PCGs were significantly lower in the NCG than in the CG. 13 PCGs were not significantly different among the FG, GG, and DG (Fig. 4).

## Discussion

Studies have revealed that the adaptive patterns of organisms are undergoing more ecological changes than previously expected^36^. Mitochondria play important roles in adaptive evolution^37^. Animals depend on the energy produced by mitochondria for locomotion and maintenance of body temperature. To adapt to environmental changes, the mitochondrial genes of bovids may be affected by selection pressure. Therefore, we focused on the evolution of Bovidae mtDNA associated with habitat type and locomotor preference and explored the evolutionary rate of mitochondrial genes in bovids at the molecular level.

### The non-synonymous/synonymous substitution rate

MtDNA PCGs play a key role in energy production; thus, assessing the selection pressure on mtDNA PCGs is important for understanding the adaptive evolution of bovids. The dN/dS is often used to determine the selection pressure on PCGs and is an effective parameter for studying the adaptive evolution of species^38,39^. In this study, the ω of mtDNA PCGs in the 48 bovids were lower than 1. Our research revealed that the Bovidae mtDNA was subjected to purifying selection in diverse habitat types and locomotor preferences. Purification selection can remove certain deleterious mutations and is essential for maintaining the function of PCGs. Some studies have also revealed that the mtDNA of marine turtles, birds, and Tibetan loaches are under purifying selection^36,40,41^. This phenomenon was associated with the presence of highly conserved mtDNA PCGs^42^. Because of the characteristics of maternal inheritance and high conservation, mitochondrial genes have become important molecular markers in the study of animal adaptive evolution.

### Selection pressure comparison of bovidae in different habitat types

ANOVA was used to detect variations in the evolutionary rate of the Bovidae in different habitat types. ANOVA revealed a marked difference in the ω of ND1 between FG and GG. ND1 is involved in the first step of OXPHOS, and mutations in ND1 are associated with diseases such as cancer^43^. The rapid evolutionary genes were not detected using the branch model in bovids inhabiting different habitat types. The results of branch-site model revealed that the positive selection sites of mtDNA PCGs differed under the influence of different habitats. Some research groups have shown that the environment drives the evolution of mtDNA PCGs in animals^44,45^. These environmental factors can be extreme, such as low temperature, low oxygen and cold. With only three categories available, the statistical noise could be too high to find an effect. Thus the rather broad categories for habitats do not necessarily reflect the situation the individuals have to face in situ. Statistical analysis indicated that diverse habitat types (forest, grassland, and desert) do not drive the evolution of mtDNA PCGs in bovids.

### Selection pressure comparison of bovidae in different locomotor preference

The Wilcoxon test was used to detect the effects of locomotor preference on Bovidae mtDNA. Our results revealed that the ω of the partial mtDNA PCGs (13 PCGs; COX1, COX2, COX3, ATP6, ND5, and Cytb) in the CG was significantly faster than that in the NCG. These genes encode subunits of the following OXPHOS proteins: cytochrome c oxidase, NADH ubiquinone oxidoreductase, ubiquinol cytochrome c oxidoreductase, and ATP synthase^46^. Mutations in the mitochondrial PCGs can affect the production of reactive oxygen^47^. Reactive oxygen species are essential for the maintenance of various functions in living organisms. Some research has revealed that the evolutionary patterns of mtDNA PCGs are associated with their locomotor abilities^40,48^. Because animals require more energy for their locomotor preferences, and energy is produced primarily in mitochondria^49^. Similarly, our results revealed that the ω of each mtDNA PCG was higher in CG than in NCG. The branch-site model revealed that a large number of positive selection sites existed in the CG. The positively selected sites in the CG might be related to adaptations to energy consumption^50^. Statistical analysis indicated that there are significant differences in ω under locomotor preference. This result is consistent with those of the branch and branch-site models. In general, the evolution of Bovidae mtDNA is driven by locomotor preference.

## Conclusions

In this study, we explored the evolution of Bovidae mtDNA in diverse habitats (forest, grassland and desert) and their locomotor preferences (climbing and non-climbing). Our results indicate that the evolution of Bovidae mtDNA is driven by locomotor preference, independent of habitat type. Our conclusions are based on the existing mitochondrial data and grouping data of bovid. The ω demonstrated that the mtDNA of the bovids had undergone purifying selection during the evolution. Our research provides valuable information that can be used for further evolutionary research on Bovidae.

### Supplementary Information

Supplementary Table S1.

Supplementary Table S2.

Supplementary Table S3.

Supplementary Table S4.

## Data Availability

All the mitochondrial genomes used in this study were accessed from the GenBank repository (https://www.ncbi.nlm.nih.gov/) under the accession numbers stipulated in Table S1.
